# Frequency and Mortality Risk Factors of Acute Ischemic Stroke in Emergency Department in Burkina Faso

**DOI:** 10.1155/2020/9745206

**Published:** 2020-06-11

**Authors:** Alfred Anselme Dabilgou, Alassane Dravé, Julie Marie Adeline Kyelem, Saïdou Ouedraogo, Christian Napon, Jean Kaboré

**Affiliations:** ^1^Department of Neurology, University Hospital Yalgado Ouedraogo, Ouagadougou, Burkina Faso; ^2^Department of Neurology, Regional University Hospital of Ouahigouya, Burkina Faso; ^3^Department of Neurology, University Hospital of Bogodogo, Ouagadougou, Burkina Faso

## Abstract

**Objective:**

To determine the prevalence of ischemic stroke deaths and their predictive factors in the Emergency Department at Yalgado Ouedraogo University Teaching Hospital (YOUTH). *Methodology*. This was a retrospective study with an analytical and descriptive focus over a period of three years from January 1, 2015, to December 31, 2017.

**Results:**

During the study period, 302 acute ischemic stroke patients with a mean age of 62.2 ± 14.26 years were included. Atrial hypertension was the most common vascular risk factor in 52.5%. On admission, 34.8% of patients had loss of consciousness. The mean time to perform brain CT was 1.5 days. The average length of stay was 4 days. Electrocardiogram, echocardiography, and cervical Doppler were not performed during hospitalization in ED. The mortality rate was 39%, respectively, 37.6% in male and 41.6% in female. The mean age of patients who died in ED was 63.6 ± 13.52 years. Hypertension was the most common vascular risk factors in 54.2% of death. After logistic regression, the predictors of death were past history of heart disease, consciousness disorders, hyperthermia, hyperglycemia on admission, poststroke pneumonia, and urinary tract infection.

**Conclusions:**

Acute ischemic stroke was frequent in Emergency Department with high mortality rate. The mortality risk factors were the same than those found in literature. This higher mortality can be avoided by early diagnosis and an adequate management.

## 1. Introduction

Stroke is the leading cause of acquired physical disability in adults, the second leading cause of dementia after Alzheimer's disease, and the third leading cause of death in developing countries [1, 2]. Stroke, in addition to the physical consequences for the individual, has a significant psychological and financial impact on the family and the health system [3]. About 85% of all stroke deaths are registered in low- and middle-income countries, which also account for 87% of total losses due to stroke in terms of disability-adjusted life years (DALYs), calculated, worldwide, in 72 million per year [4]. Demographic changes and inadequate control of major risk factors such as high blood pressure, heart disease, obesity, diabetes, and smoking add to the burden of this condition in sub-Saharan Africa [5, 6]. The prognosis in patients with ischemic stroke may be improved by thrombolysis associated with statin therapy [7]. In African setting, there is no prehospital care for acute stroke patients and thrombolysis is not available. The short-term mortality of stroke was elevated according to a study from Ghana which had showed that 69% of stroke patients died within 24 hours of the onset of stroke [8]. In Burkina Faso, there are few data concerning the short-term mortality of ischemic stroke. The aim of this study was to determine the frequency and mortality risk factors of ischemic stroke in Emergency Department in order to improve the survival of patients in acute phase of stroke.

## 2. Methodology

### 2.1. Location of the Study

Burkina Faso is a French speaking country located in West Africa region. 224,000 km^2^ with a population of 16,000,000 inhabitants according to 2016 National Census. The city of Ouagadougou has four university hospitals for a population of 3 million. Yalgado Ouedraogo University Teaching Hospital is one of the 3 main tertiary hospitals taking care of neurological disorders. All the patients with suspected diagnosis of ischemic stroke were admitted first in the Emergency Department and after transferred in the Resuscitation Department or the Neurology Department for management. The hospital has a 24-hour scanning service. Basic biological investigations (WBC, CRP, electrolytes tests, Rhesus blood group, and blood sugar) are carried out urgently at the hospital.

### 2.2. Study Design

We conducted a 3-year cross-sectional study from January 1, 2015, to December 31, 2017, at the Emergency Department of Yalgado Ouedraogo University Hospital.

### 2.3. Study Population

We recorded the data of ischemic stroke patients aged over 15 years admitted at the Emergency Department of YOUTH during the period from January 1, 2015, to December 31, 2017. We excluded patients who had Transient Ischemic Attack and those whose information was not available.

### 2.4. Data Analysis

The variables assessed in this study include sociodemographic data (age, sex, residence, marital status, and occupation), clinical data (vascular risk factors, admission time, body temperature, blood pressure, state of consciousness, and NIHSS score), investigations (time to perform brain CT, fasting blood glucose, blood count, electrolytes test, and renal balance), treatment (antiaggregant agents, antihypertensive drug, insulin, etc.), and stroke outcome (length of stay, complications, mRS, and mortality rate).

The data were collected from medical records of patients, hospitalization register of patients, and treatment and monitoring sheets for patients. For analysis, Epi info version 7.1.5.2 was used. Continuous variables were compared using Student's *t*-test for normally distributed variables, and categorical variables were compared using a chi-squared (*χ*^2^) test. A bivariate analysis was first performed to evaluate the association between stroke mortality and sociodemographic variables (age <65 years, gender, and location of patients), vascular risk factors (hypertension, diabetes mellitus, heart disease, previous stroke, smoking, and alcohol), delay of arrival in 24 hours, Glasgow conscience score, time to perform CT in 24 hours, hyperglycemia, anemia, renal failure, poststroke complications (pneumonia, urinary tract infection, and cutaneous infections), and length of stay in 7 days. Multivariate analysis was performed to determine the independent mortality risk factors. The test is considered statistically significant for *p* less than 0.05.

### 2.5. Ethical and Deontological Considerations

Each patient admitted in a YOUTH department had a medical record in this department during their admission. Patient data was manually archived in the form of medical records, hospitalization, and discharge registers. For this study, we had the authorization of local ethics committee of the hospital and ethics board of the medical school of University Joseph Ki-Zerbo of Ouagadougou. The privacy of the patients and the confidentiality of the data collected have been complied with.

### 2.6. Assessments

Stroke is defined as “the rapid development of localized or global clinical signs of cerebral dysfunction with symptoms lasting more than 24 hours that may lead to death, with no apparent cause other than vascular origin,” according to the report WHO [9].

Lacunar infarct is classically defined as a small deep-seated cerebral infarction, the mechanism of which is an occlusion of a small intracranial perforating artery associated with hypertensive microangiopathy. Hyperglycemia defined as case-specific blood glucose greater than 6.1 mmol/l (sup 1.26 g/l) during acute hospitalization. Hypoglycemia was defined by a blood glucose lower than 4 mmol/l (0.7 g/l). Anemia was defined as hemoglobin level less than 12 g/dl in women or less than 13 g/dl in men. Severe anemia was defined when hemoglobin level is less than 6 g/dl. Hypo- and hypernatremia were defined by a level of atresia <136 mmol/l and >145 mmol/l, respectively.

Hypo- and hyperkalemia were defined by a level of potassium <3.3 mmol/l and >5.0 mmol/l, respectively. Hypo- and hypercalcemia were defined by a level of calcemia <32.2 mmol/l and >32.65 mmol/l, respectively.

## 3. Results

### 3.1. Characteristics of Study Population

During the study period, 2169 patients were admitted in the Emergency Department for stroke, out of which 1325 (61%) were ischemic. Our study had included 302 (22.8%) ischemic stroke patients who had available and complete information. Two hundred and fourteen (76.2%) had been referred by health center to ED, and the remaining (23.8%) were directly admitted from their houses. The mean time between admission and disease onset was 2 days (range: 1-12 days). One hundred sixty-seven (55.3%) were admitted within the first 24 hours, 97 (32.1%) between 24 and 48 hours, and remaining 38 (12.6%) after 48 hours. One hundred and five (44.7%) patients arrived within the delay of 4 h 30 mn. The majority of the patients was male gender (62.1%) with a mean age of 62.7 ± 14.26 years (range: 26-90 years). The mean age of men and women was, respectively, 62.1 ± 14.45 years and 63.7 ± 14.46 years. The median age was represented by the age group of 65-75 years (34%). According to occupation, 38.5% of the patients were farmers; 25.5%, housewives; and 14%, traders. Residence of the patients was indeterminated in 2 (0.7%). The marital status was specified in 222 (73.5%) patients; among them, 186 patients (83.8%) were married and 36 (16.2%), single. Atrial hypertension was the most common vascular risk factor in 52.5% (*n* = 156), followed by alcohol consumption in 20.9% (*n* = 63). The most admission pattern was motor deficit (78.8%), followed by aphasia (31%) and disorders of consciousness (22.3%). On admission, 55 (18.2%) patients had hyperthermia (≥38.5°C). Ninety six (34.8%) patients had GCS <8; 24, between 8 and 12; and 65, between 13 and 14. Hemiplegia was the most common neurological disorders (74.6%), followed by aphasia (31%). The mean time to perform brain CT was 1.5 days (range: 1-4 days). One hundred and seventy-four (58%) patients had performed it within the first 24 hours of admission. The most location of ischemic stroke lesions was the middle cerebral artery in 71.6% (*n* = 235), anterior cerebral artery in 22.8% (*n* = 69), posterior circulation stroke in 5.9% (*n* = 18), and anterior choroidal artery in 2% (*n* = 6). The other investigations available in ED were blood glucose in 290 (96%) patients, blood cell count in 272 (90.1%), renal function in 217 (71.9%), and electrolytes tests in 207 (68.5%). Hyperglycemia was found in 16.2% (*n* = 47) and hypoglycemia, in 1% (*n* = 3) of cases. Anemia was found in 11.8% (*n* = 32) of cases. The most electrolyte disorders were hypocalcaemia in 14.4% (*n* = 29), followed by hypokalemia in 10.4% (*n* = 20) and hypernatremia in 7.5% (*n* = 14). Electrocardiogram, echocardiography, and cervical Doppler were not performed during hospitalization in ED. Antiplatelets were prescribed in 295 (98.4%) patients; antihypertensive agents, in 185 (61.7%); and statins, in 114 (38.13%) patients. Mechanic thrombectomy and thrombolysis were not available in Burkina Faso. The average length of stay was 4 days (range: 1-14 days). The most common medical complication during hospital admission was pneumonia present in 75 (24.8%) of patients, followed by urinary tract infections (18; 6%) and cutaneous infections (8; 2.6%).

### 3.2. Mortality and Mortality Risk Factors

Death was recorded in 118 (39.1%) patients. The mortality was, respectively, 31% within 7 days and 39.3% within 14 days. Out of which, 79.7% (*n* = 94) occurred in 7 days and the rest 20.3% (*n* = 24), in 7-14 days. The majority of dead patients was male (60.1%). The mortality rate was 37.6% in male and 41.6% in female. The mean age of dead patients was 63.6 ± 13.52 years. The mean age of men and women was, respectively, 64.4 and 62.3 years (*p* = 0.281). Hypertension was the most common vascular risk factors in 54.2% of death (*n* = 64). Sixty-two (52.5%) of the dead patients were admitted in the first 24 hours after the disease symptom. Forty-two (35.6%) of the dead patients had fever, and 74 (62.7%) of them had GCS <10. Brain CT was performed after 24 hours of admission in 74 (62.7%) of the dead patients. Biological assessment found that stress hyperglycemia and anemia were present, respectively, in 78 (66.1%) and 17 (14.4%) of the dead patients. Poststroke pneumonia, cutaneous infections, and urinary tract infections were seen, respectively, in 57 (48.3%), 23 (19.5%), and 15 (12.7%) of the dead patients in ED. After the bivariate analysis, the predictors of death were history of heart disease (*p* = 0.048), consciousness disturbance on admission (*p* ≤ 0.001), fever (*p* = 0.01), delayed completion of CT scan greater than 24 hours (*p* ≤ 0.001), hyperglycemia (*p* = 0.002), anemia (*p* = 0.038), poststroke pneumonia (*p* ≤ 0.001), and urinary tract infection (*p* ≤ 0.001). The length of stay was under 7 days in 92 (78%) of the dead patients (*p* = 0.384).  Table 1 gives the characteristic of acute ischemic stroke patients admitted in ED. After multivariate logistic regression analysis, the predictors of death were history of heart disease (*p* = 0.031), consciousness disorders (*p* = 0.031), hyperthermia (*p* = 0.004), hyperglycemia (0.008), poststroke pneumonia (*p* ≤ 0.001), and urinary tract infection (*p* = 0.007).  Table 2 shows the results of multivariate logistic regression analysis.

## 4. Discussion

### 4.1. Mortality Rate

This single hospital study focused on short-term mortality of ischemic stroke and its risk factors in LMICs. The mortality rate for ischemic stroke in our study (38.1%) was higher than that in the study of Diop-Sène et al. in Senegal (20.4%) [10] and the study of Martins et al. in Brazil (10.2%) [11]. Our mortality was similar to those observed in ICU (37.8%) where patients with severe stroke were admitted [12]. According to literature, the mortality in neurology wards varied between 5% and 15% with a rate of around 5% in patients that were initially treated at a stroke unit [13, 14]. The higher mortality in context could be explained by the fact that we did not have stroke unit, intensive care unit, or thrombolysis treatment for the whole country and capacities of Emergency Department were limited. Recently, early case fatality was implemented as an indicator to measure early stroke mortality, because most important clinical decisions are made in the first week after hospital admission [15]. The early mortality in our study (31.1%) was similar to those observed in African context, in Ghana (32.7%) [16]. In contrast, the 7-day mortality was lower in western countries where stroke unit is available (2%) [17]. The majority of stroke mortality (79.7%) occurred in the first 7 days, in line with Heuschman et al. (66%) [18] but in contrast with Das et al. who had found that 50% of case fatality occurred in 7-14 days [19]. Early case fatality was related to neurologic death (other medical conditions) and was therefore primarily influenced by factors such as initial stroke severity and early neurologic deterioration [18].

### 4.2. Characteristics of Dead Patients

According to sociodemographic characteristics, the mean age of the dead patients in ED was 63.6 ± 13.52 years, lower than in Turkey (70.7 ± 12.9 years) [14]. The difference between these two studies was due to the life expectancy which had more elevated in Turkey than in Burkina Faso. The majority of dead patients were male (60.1%), in concordance with the studies of Moon et al. in Senegal [15] and Agyemangi et al. in Nepal [16]. In contrast, Koennecke et al. in Turkey had found a majority of female patients in 55.6% [14]. The male predominance reported could be explained by the higher frequency high risk factors found in men such as tobacco and alcohol. According to sex mortality, mortality rate in women (41.6%) was higher than that in male (37.6%). This finding is comparable with those of previous studies [23, 24] but in contrast with the study of Diop-Sène et al. in Ghana with a majority of male patients (43.4%) [10]. Women had some difficulties to access to medical ward and had late arrival in ED. In terms of mortality, male patients were more older than female (64.4 years versus 62.3 years), similar in Nepal whom the age was 77.12 ± 6.37 years in male and 65 ± 7.2 years in female [16]. According to vascular risk factors, the majority of dead patients were hypertensive (50.8%), similar in the study of Touré et al. in Senegal (69.7%) [21] and Koennecke et al. in Turkey (77.8%) [14]. According to literature, hypertension is a leading cause of death [20]. The majority of patients had late arrival at hospital (55.3%), after the delay of 4 h 30 mn. Khan et al. had showed that only 13.4% of the patients reached the hospital within 3 hours [26]. Schellinger et al. had found that 44.4% of the patients referring to the hospital arrived more than three hours later [27]. In contrast, most studies indicate that a vast majority of patients referring to the hospital reached within the first three hours of the onset of symptoms [28]. The late arrival of patients at Emergency Department could be explained by the majority of them who was transported by nonmedicalized ambulance or taxis. Patients coming from rural areas were transported by nonmedicalized ambulance. No patients did attend traditional healers first. Our study did not found significant link between stroke mortality and late arrival after 24 hours. These findings were similar in the study of Khan et al. who found no significance between stroke mortality and delay of arrival within 3 hours [26].

### 4.3. Mortality Risk Factors

Our study had identified that performing brain CT after 24 hours was a mortality risk factor for ischemic stroke patients admitted in the Emergency Department. We are not aware of a previous study that looked specifically at the association between time to perform brain CT on admission and in-hospital mortality for stroke in sub-Saharan Africa. Lekoubou et al. in Cameroon had found that no CT performed on admission was predictor of mortality for ischemic or hemorrhagic stroke patients [29]. According to recommendations, a suspected stroke patient should have a CT within 3 hours of symptom onset to allow for appropriate intervention to arrest progression of neurological deficits [30]. The majority of ischemic stroke patients (58%) had performed brain CT within the first 24 hours of admission. This situation was greater in dead patients in whom 62.7% had performed brain CT after 24 hours of stroke onset. For example, in the United States of America, plus 90% of ischemic patients had brain imaging within 24 hours of stroke outcome [31]. This late presentation to brain CT could be explained by the fact that CT is not available widely, particularly for patients living in rural areas (38.7% of patients in our case). Second, 52.5% of the dead patients were admitted in the first 24 hours after disease symptom due financial constraints (housewives and cultivators). Maestroni et al. found that patients presenting within 3 h were more likely to have a CT earlier than patients arriving after 3 h [32]. Anemia was reported in 11.8% of patients with ischemic, in consistent with literature (6-40%) [33]. It was identified as nonindependent mortality risk factors in our study, in line with several studies which had found that both low and high hemoglobin levels are associated with increased mortality [34, 35]. Other studies had found no association between anemia and stroke outcome [36, 37]. We had showed that patients with past history of cardiopathy had more risk of mortality than others. Most of them were hypertensive with irregular follow-up due to high cost of antihypertensive agents. In a study from Turkey, Ceylan et al. had demonstrated that mortality was high in patients with coronary artery disease history (*p* = 0.03) and ejection fraction of the dead patients was lower than the others (*p* = 0.002) [20]. History of cardiopathy was associated to cardioembolism and higher mortality. Due to the emergency, cardiovascular investigations (electrocardiogram, cardiac echo-Doppler, carotid echo-Doppler, angioscanner) were not carried out. The other relevant independent factor associated with short-term mortality in ischemic stroke patients (consciousness disturbance, stress hyperglycemia, fever, poststroke pneumonia, and urinary tract infection) were well known in literature. Consciousness disturbance is correlated with high mortality rate [38–40]. For Touré et al. in Senegal [21], conscience disorders multipled per 3.75 time the mortality rate. Concerning fever, Garbusinski et al. [41] had identified fever as mortality risk factor of ischemic stroke in Gambia. We had found the same result in our study.. While stress hyperglycemia was present in a fair proportion of dead patients (23.8%), it was also an independent mortality risk factor for ischemic stroke, in concordance with several studies [21, 25, 42]. Poststroke pneumonia was rare (24.8%) but was an independent mortality risk factor of death for ischemic stroke patients admitted in ED. For Katzan et al., pneumonia has been associated with a relative risk of 3.0 for mortality in a study including 14,293 acute stroke patients [43]. Urinary tract infection had been identified as independent mortality risk factor (OR = 9.33), in line with the study of Walker et al. in Gambia [3]. According to Willeke, pneumonia and urinary tract infection both increase the risk for unfavorable outcome and pneumonia is associated with mortality with an OR of 3.62 [44].

### 4.4. Strengths and Limitations of the Study

This is the first study of its kind carried out on the early mortality of ischemic stroke in Burkina Faso. However, this study had several limitations due to its retrospective characteristics and small sample. In fact, several patient files were missing and some data could not be updated. So, we did not generalize the results to all ischemic stroke patients admitted in the Emergency Department. Our study was unable to assess the impact of etiology and thrombolysis treatment on early mortality from ischemic stroke.

## 5. Conclusions

Acute ischemic stroke was frequent in the Emergency Department. The early mortality of acute ischemic stroke was higher than that in developed countries but similar to that observed in African context. Apart from history of cardiopathy and large delay of head CT, we noticed the same mortality factors than in literature.

## Figures and Tables

**Table 1:** Characteristic of acute ischemic stroke patients admitted in ED.

Variables	Died (*n* = 118)	Discharges (*n* = 184)	OR	*p* value
Age (years)				
>65	47 (35.4%)	86 (64.6%)		
<65	71 (42%)	98 (58%)	1.33	0.238
Gender				
Male	71 (37.4%)	119 (62.6%)		
Female	47 (42%)	65 (58%)	1.21	0.429
Location				
Urban	60 (32.8%)	123 (67.2%)		
Rural	54 (40.1%)	63 (53.9%)	0.844	0.476
Hypertension				
Yes	64 (40.5)	92 (59.5)		
No	54 (37%)	91 (67%)	1.16	0.429
Diabetes mellitus				
Yes	10 (52.6%)	09 (47.4%)		
No	108 (38.3%)	174 (61.7%)	1.79	0.215
Heart disease				
Yes	10 (62.5%)	06 (37.5%)		
No	108 (38.3%)	174 (61.7%)	2.75	0.048
Previous stroke				
Yes	12 (46.1%)	14 (53.9%)		
No	106 (46.1%)	170 (53.9%)	1.37	0.439
Smoking				
Yes	10 (40.6%)	26 (59.4%)		
No	108 (27.8%)	158 (72.2%)	0.562	0.139
Alcohol				
Yes	18 (29.6%)	45 (74.4%)		
No	100 (41.8%)	139 (58.2%)	0.556	0.056
Delay of arrival (hours)				
≤24	62 (45.9%)	73 (54.1%)		
>24	56 (49.1%)	58 (50.9%)	0.7964	0.376
(GCS < 10)				
Yes	74 (70.5%)	31 (29.5%)	8.3	
No	44 (22.3%)	153 (77.7%)		≤0.001
Fever				
Yes	42 (76.4%)	13 (23.6%)		
No	76 (30.8%)	171 (69.2%)	7.269	0.01
Brain CT (hours)				
≤24	44 (25.3%)	130 (74.7%)		
>24	74 (54.8%)	54 (42.2%)	2.337	≤0.001
Hyperglycemia				
Yes	27 (57.5%)	21 (42.5%)		
No	91 (38.1%)	148 (61.9%)	2.22	0.002
Anemia				
Yes	17 (53.1%)	15 (46.9%)		
No	101 (42.1%)	139 (57.9%)	5.833	0.038
Renal failure				
Yes	08 (50%)	08 (50%)		
No	110 (54.7%)	91 (45.3%)	1.644	0.335
Poststroke pneumonia				
Yes	57 (76%)	18 (24%)		
No	61 (26.9%)	166 (73.1%)	9.333	≤0.001
Urinary tract infection				
Yes	15 (83.3%)	03 (16.7%)	9.337	≤0.001
No	103 (36.3%)	181 (63.7%)		
Cutaneous infections				
Yes	2 (22.2%)	7 (77.7%)		
No	116 (39.6%)	177 (60.4%)	1.311	0.384
Length of stay (days)				
≤7	92 (38.2%)	149 (61.8%)		
>7	26 (42.6%)	35 (57.4%)	1.311	0.384

**Table 2:** Results of multivariate analysis.

Covariables	Odds ratio	95% CI	*p* value	Significance
Age (<65 and ≥65 years)	1.97	0.831-4.682	0.123	^∗^
Gender (M/F)	0.908	0.71-2.224	0.834	^∗^
Hypertension (Y/N)	1.25	0.502-31.56	0.623	^∗^
Diabetes mellitus (Y/N)	2.023	0.406-10.084	0.39	^∗^
Heart disease (Y/N)	6.72	1.186-38.071	0.031	+
Previous stroke (Y/N)	0.603	0.124-2.92	0.53	^∗^
Smoking (Y/N)	0.975	0.189-5.017	0.977	^∗^
Alcohol (Y/N)	0.449	0.125-1.607	0.213	^∗^
Consciousness disorders (Y/N)	2.692	0.59-12.28	0.031	+
Fever (Y/N)	5.06	1.663-4.353	0.004	+
Time to perform brain CT >24 h (Y/N)	1.762	0.713-4.353	0.219	^∗^
Hyperglycemia (Y/N)	6.503	0.86-8.991	0.008	+
Anemia (O/N)	3.792	0.276-52.048	0.319	^∗^
Renal failure (normal/altered)	1.08	0.191-6.098	0.93	^∗^
Poststroke pneumonia (Y/N)	9.528	3.253-27.904	≤0.001	+
Urinary tract infection (Y/N)	15.08	2.09-108.813	0.007	+
Cutaneous infections (Y/N)	7.949	0.235-268.37	0.248	^∗^
Length of stay (<7 days and ≥7 days)	1.246	0.355-4.364	0.731	^∗^

+: positive impact; ^∗^: no impact.

## Abbreviations

DALYs: Disability-adjusted life years
YOUTH: Yalgado Ouedraogo University Teaching Hospital
WHO: World Health Organization
CT: Computed tomography
LMICs: Low- and middle-income countries
ED: Emergency Department
GCS: Glasgow Coma Scale
OR: Odds ratio.
